# Supplementary material for: Content-rich biological network constructed by mining PubMed abstracts
Source: BMC Bioinformatics. 2004 Oct 8;5:147. doi: 10.1186/1471-2105-5-147 (PMC528731; doi:10.1186/1471-2105-5-147)
Supplement: Additional File 2 — The original results of the above study (non-essential files are deleted to keep the file size under the limit set by BMC bioinformatics). [file 1471-2105-5-147-S2.bz2 › chilibotAdditionalFile2/dip05/392ID8700872E9878/html/FLT1_PGF.html]

 


 **FLT1** and **PGF** 
  
Found 101 abstracts in PubMed, retrieved 05.  
 

 What does Google say? 
 PDF only 
| .edu only 

---

**Interactive relationship** (e.g. stimulation, inhibition, etc)

**Inhibitory relationship**- Placental growth factor  [ **PGF** ]  1 PlGF  [ **PGF** ]  1, which exclusively binds VEGFR 1  [ **FLT1** ] , decreased hyperoxia induced retinal vaso obliteration from 22.2% to 5.1%.  Ref: 12840058 J Clin Invest, 2003
**Neutral relationship**- PlGF  [ **PGF** ]  promotes recruitment of VEGFR1  [ **FLT1** ]  HSCs from a quiescent to a proliferative microenvironment within the BM, favoring differentiation, mobilization, and reconstitution of hematopoiesis.  Ref: 12799282 Ann N Y Acad Sci< MedlineTA>Ann N Y Acad Sci, 2003
- In this review, we describe the molecular properties of PlGF  [ **PGF** ]  and VEGFR 1  [ **FLT1** ]  and how this translates into an important role for PlGF  [ **PGF** ]  in the angiogenic switch in pathological angiogenesis, by interacting with VEGFR 1  [ **FLT1** ]  and synergizing with VEGF.  Ref: 12871269 J Thromb Haemost, 2003
- They belong to the larger family which also includes VEGF, placenta growth factor  [ **PGF** ]  PlGF  [ **PGF** ]  and VEGF B, VEGF C and VEGF D are ligands for the endothelial cell specific tyrosine kinase receptors VEGFR  [ **FLT1** ]  2 and VEGFR  [ **FLT1** ]  3.  Ref: 12888864 Thromb Haemost, 2003

**Non-interactive relationship** (e.g. studied together, co-existance, homology, etc.)

- Placental growth factor  [ **PGF** ]  and its receptor, vascular endothelial growth factor receptor 1  [ **FLT1** ]  novel targets for stimulation of ischemic tissue revascularization and inhibition of angiogenic and inflammatory disorders.  Ref: 12871269 J Thromb Haemost, 2003
- The mechanism whereby PlGF  [ **PGF** ]  enhanced early phases of BM recovery was mediated directly through rapid chemotaxis of readily available VEGFR1  [ **FLT1** ]  BM repopulating and progenitor cells.  Ref: 12799282 Ann N Y Acad Sci< MedlineTA>Ann N Y Acad Sci, 2003
- In contrast to VEGF and its receptor VEGFR  [ **FLT1** ]  2, PlGF  [ **PGF** ]  and its receptor VEGFR 1  [ **FLT1** ]  have been largely neglected and therefore their potential for therapy has not been previously explored.  Ref: 12871269 J Thromb Haemost, 2003
- Plasma elevation of placental growth factor  [ **PGF** ]  PlGF  [ **PGF** ] , which signals through VEGFR1  [ **FLT1** ] , but not VEGFR2, restored hematopoiesis during the early and late phases following BM suppression.  Ref: 12799282 Ann N Y Acad Sci< MedlineTA>Ann N Y Acad Sci, 2003
- activation of VEGFR 1  [ **FLT1** ]  by PlGF  [ **PGF** ]  1 is a selective strategy for preventing oxygen induced retinal ischemia without provoking retinal neovascularization.  Ref: 12840058 J Clin Invest, 2003
- Thus, these preclinical studies show proof of principle that PlGF  [ **PGF** ]  and VEGFR 1  [ **FLT1** ]  are promising therapeutic targets to treat angiogenesis and inflammation related disorders.  Ref: 12871269 J Thromb Haemost, 2003
